# Muscle metastases: comparison of features in different primary tumours

**DOI:** 10.1186/1470-7330-14-21

**Published:** 2014-05-06

**Authors:** Alexey Surov, Johanne Köhler, Andreas Wienke, Hubert Gufler, Andreas Gunter Bach, Dominik Schramm, Curd Behrmann, Rolf Peter Spielmann

**Affiliations:** 1 Department of Radiology, Martin-Luther-University Halle-Wittenberg, Ernst-Grube-Str. 40, 06097 Halle, Germany; 2 Department of Epidemiology, Biometry and Informatics, Martin-Luther-University Halle-Wittenberg, Ernst-Grube-Str. 40, 06097 Halle, Germany; 3 Department of Biometry and Medical Statistics, Martin-Luther-University Halle-Wittenberg, Ernst-Grube-Str. 40, 06097 Halle, Germany

**Keywords:** Muscle metastases, Radiological features, Computed tomography, Magnetic resonance imaging, Primary tumours

## Abstract

**Background:**

Muscle metastases (MM) from solid tumours are rare. The aim of this study was to describe radiological features of MM, and to compare their patterns in different malignancies.

**Methods:**

A retrospective search in the statistical database of our institution revealed 61 cases of MM. Additionally, a retrospective search in Pubmed database was performed. Together with our cases the present analysis comprises 461 patients (682 MM).

**Results:**

MM derived from the following malignancies: lung cancer (25.1%), gastrointestinal tumours (21.0%), and urological tumours (13.2%). Other neoplasias with MM were rare. MM were localised most frequently in the thigh muscles, the extraocular musculature, and the gluteal and paravertebral muscles. The localisation of MM was different in several primary malignancies.

On computed tomography (CT), five different patterns of MM occurred: masses with homogeneous contrast enhancement (type I, 46.5%), abscess-like lesions (type II, 27.7%), diffuse infiltration with muscle swelling (type III, 18.1%), intramuscular calcifications (type IV, 6.5%), or MM presented as intramuscular bleeding (type V, 1.2%). MM from several primary tumours manifested with different CT patterns.

On MRI, most MM were hyperintense in comparison to unaffected musculature in T2 weighted images and hypo- to isointense on T1 weighted images with a heterogeneous enhancement. There were no differences in MRI features of MM in different primary tumours. On ultrasound, most MM were hypoechoic. On positron emission tomography, MM presented as focally abnormal intramuscular uptake.

**Conclusion:**

MM present with a broad spectrum of radiological features. Different CT imaging findings of MM were observed in different primary tumours. The localisation of MM also varies with different primary malignancies.

## Background

Muscle metastases (MM) from solid tumours are rare. The prevalence of MM varies from 0.03% to 5.6% in autopsy series [1-4], and from 1.2% to 1.8% in radiological series [5,6].

This is due to the fact that muscles have several protective mechanisms against metastatic invasion [4,7]. According to the literature, the musculature produces several biochemical anti-tumour factors, and it can damage tumour cells biomechanically [7-10]. Previously, several radiological features of MM were reported [6,11-13]. According to Pretorius and Fishman, the most common appearance of MM on computed tomography (CT) was an isolated intramuscular mass with central low attenuation and rim enhancement [14]. However, in other reports, masses with homogeneous enhancement were the most frequent pattern of MM [6]. In addition, other imaging features, such as intramuscular calcifications, muscle infiltration and muscle bleeding were also documented [6,11,12,15].

As reported previously, on magnetic resonance imaging (MRI), MM were hypointense on T1 weighted (T1w) images and hyperintense on T2 weighted (T2w) images, with marked enhancement after contrast administration [5,12,15,16]. However, hyperintense lesions on T1w images and slightly enhancing lesions have also been described in the literature [17].

It must be presumed that radiological patterns of MM vary in different primary tumours. However, up to now, it was not examined whether some entities are more likely to cause a certain radiological pattern of MM than others.

Therefore, the purpose of this study was to describe radiological features of muscle metastases, and to compare their patterns in different malignancies.

## Methods

### Patients and literature review

A retrospective search in the statistical database of our institution from January 2000 to December 2007 revealed 61 cases of MM from solid malignancies.

Additionally, a retrospective search in Pubmed database using the keywords “muscle metastasis”, “muscle metastases”, “intramuscular metastasis”, “intramuscular metastases” and “metastases to the musculature” was performed. Publications in the time interval from 1990 to 2010 were considered. Secondary references were also reviewed.

Inclusion criterion for MM lesions was a sufficient description of CT, and/or MRI, and/or sonographic and/or PET features.

After thorough analysis 274 articles with 400 patients were involved in the study.

Therefore, together with our 61 cases the present analysis comprises 461 patients.

### Imaging

#### CT

In our institution 61 patients with MM were found retrospectively. In all cases CT (Somatom Plus 4 VZ, and Somatom Sensation 64, Siemens, Erlangen, Germany) was performed after intravenous application of 60–140 ml of iodinated intravenous contrast medium at a rate of 1.5-3.5 ml/s by a power injector (Medtron GmbH, Germany), with a scan delay of 30–90 s after onset of injection.

In the literature, CT findings of MM were available for 199 patients. Therefore, our analysis included CT findings of MM in 260 patients.

#### MRI

In our institution, 28 patients with MM were investigated by MRI. MR imaging was performed using a 1.5 T MRI scanner (Magnetom Vision Sonata Upgrade, Siemens, Germany). Several different scanning protocols were used depending on lesion localisation. MRI sequences included T2 weighted (T2w) images, fat-supressed T2w images and T1 weighted (T1w) images.

In 24 patients MR images were repeated after intravenous administration of contrast medium (gadopentate dimeglumine, Magnevist, Bayer Schering Pharma, Leverkusen, Germany), 0.1 ml per kilogram of body weight.

70 cases with MM were acquired from the literature. Therefore, our analysis included MRI findings of MM in 98 patients.

#### Ultrasound

Ultrasound was performed in one patent with MM in our clinic. In the literature 39 cases of MM investigated by US were reported.

#### PET and PET/CT

PET and PET/CT were not performed in our institution. PET features of 28 patients with MM were acquired from the literature.

### Statistics

Statistical analysis was performed using SPSS statistical software package (SPSS 17.0, SPSS Inc., Chicago IL, USA). Collected data were evaluated by means of descriptive statistics (absolute and relative frequencies). Continuous variables were expressed as mean ± standard deviation (SD), and categorical variables as percentages. Numbers of events between groups were compared with a chi-square test. Significance level was chosen to be 0.05.

## Results

### Primary tumours and localisation of MM

Our analysis comprises 461 patients. In these patients 682 MM were detected.

The muscle metastases derived from the following malignancies: lung cancer (25.1%), gastrointestinal tumours (21.0%), urological tumours (13.2%), genital tumours (9.3%), and breast cancer (8.2%). Other neoplasias with MM were rare (Table 1).

**Table 1:** Primary malignancies

**Tumours**		**n**	**%**
**Lung cancer**		116	**25,2**
**Gastrointestinal tumours**	small bowel carcinoma	1	**21.0**
	gall bladder carcinoma	5	
	colonic cancer	36	
	liver malignancies	7	
	stomach cancer	25	
	esophageal cancer	18	
	carcinoma of pancreas	5	
**Urological tumours**	renal cell carcinoma	38	**13,2**
	urothel carcinoma	23	
**Genital tumours**	seminoma	3	**9,3**
	ovarian cancer	7	
	prostatic cancer	4	
	endometrial carcinoma	9	
	vulvic cancer	2	
	carcinoma of cervix uteri	18	
**Breast carcinoma**		38	**8,2**
**CUP**		28	**6,1**
**Sarcoma**		22	**4,8**
**Malignant melanoma**		16	
**Carcinoma of cutis**		2	
**Thyroid gland carcinoma**		17	**3,7**
**Others**	carcinoma of larynx	4	**2,8**
	neuroblastoma	2	
	pleural mesothelioma	3	
	parotid gland carcinoma	1	
	carcinoma of pharynx	2	
	tongue carcinoma	1	
**Carcinoid**		8	**1,7**

MM were multiple in 111 (24.1%) patients and solitary in 350 cases (75.9%).

MM were localised most frequently in the thigh muscles, the extraocular musculature, and the gluteal and paravertebral muscles (Table 2).

**Table 2:** Localisation of the identified MM

**Localisation**	**n**	**%**
Thigh muscles	151	**22.1**
Extraokular muscles	102	**15.0**
Gluteal muscles	73	**10.7**
Paravertebral musculature	70	**10.3**
Iliopsoas muscle	69	**10.1**
Thoracal muscles	58	**8.5**
Upper arm muscles	50	**7.3**
Abdominal wall muscles	30	**4.4**
Lower leg musculature	29	**4.3**
Head and neck muscles	26	**3.8**
Fore arm muscles	24	**3.5**

Breast cancer metastasised more often into the extraocular musculature in comparison to other tumours (Table 3). MM from lung cancer were localised frequently in the upper and lower extremities, colorectal carcinomas metastasised often into the abdominal wall musculature, urothel carcinomas into the iliopsoas muscle, and gastric cancer into the gluteal and lower extremities muscles (Table 3).

**Table 3:** Localisation of MM in frequent different primary tumours (more than 30 lesions per tumour)

**Primary tumours**	**Localisation, n (%)**								
	**AWM**	**EoM**	**Glu**	**IP**	**HN**	**UE**	**PvM**	**Tho**	**LE**
Lung cancer, LC (n = 162)	4 (2.5)	4 (2.5) vs BC p = 0.021	12 (7.4) vs EC p = 0.042	13 (8.0)	1 (0.6)	34 (21.0) vs BC p = 0.005	20 (12.3)	24 (14.8)	50 (30.9) vs BC p = 0.005
Colorectal carcinoma, CC (n = 48)	7 (14.6) vs LC p = 0.021	1 (2.1) vs BC p = 0.021	7 (14.6)	7 (14.6)	1 (2.1)	3 (6.3)	8 (16.7)	2 (4.2)	12 (25.0) vs BC p = 0.009
Stomach cancer, SC (n = 33)	1 (3.0)	4 (12.1) vs BC p = 0.021	6 (18.2)	2 (6.1)	1 (3.0)	1 (3.0)	2 (6.1)	3 (9.1)	13 (39.4) vs BC p = 0.005
Esophageal cancer, EC (n = 46)	2 (4.3)	1 (2.2) vs BC p = 0.021	12 (26.1) vs BC p = 0.005	4 (8.7)	4 (8.7)	5 (10.9)	3 (6.5)	4 (8.7)	11 (23.4) vs BC p = 0.018
Renal cell cancer, RCC (n = 46)		4 (8.7) vs BC p = 0.021	3 (6.5)	6 (13.0)	2 (4.3)	6 (13.0)	5 (10.9)	4 (8.7)	16 (34.8) vs BC p = 0.005
Urothel carcinoma, UC (n = 37)	2 (5.4)		4 (10.8)	11 (29.7) vs BC p = 0.005		2 (5.4)	3 (8.1)	1 (2.7)	14 (37.8) vs BC p = 0.005
Breast cancer, BC (n = 73)	2 (2.7)	53 (72.6)	2 (2.7)	1 (1.4)	7 (9.6) vs BC p = 0.009	2 (2.7)	3 (4.1)		3 (4.1)

AWM abdominal wall muscles, EoM extraocular muscles, Glu gluteal muscles, IP iliopsoas muscle, HN head and neck muscles, UE upper extremities muscles, PvM paravertebral muscles, Tho thoracic wall muscles, LE lower extremities muscles.

### CT features of MM

**Figure 1:**
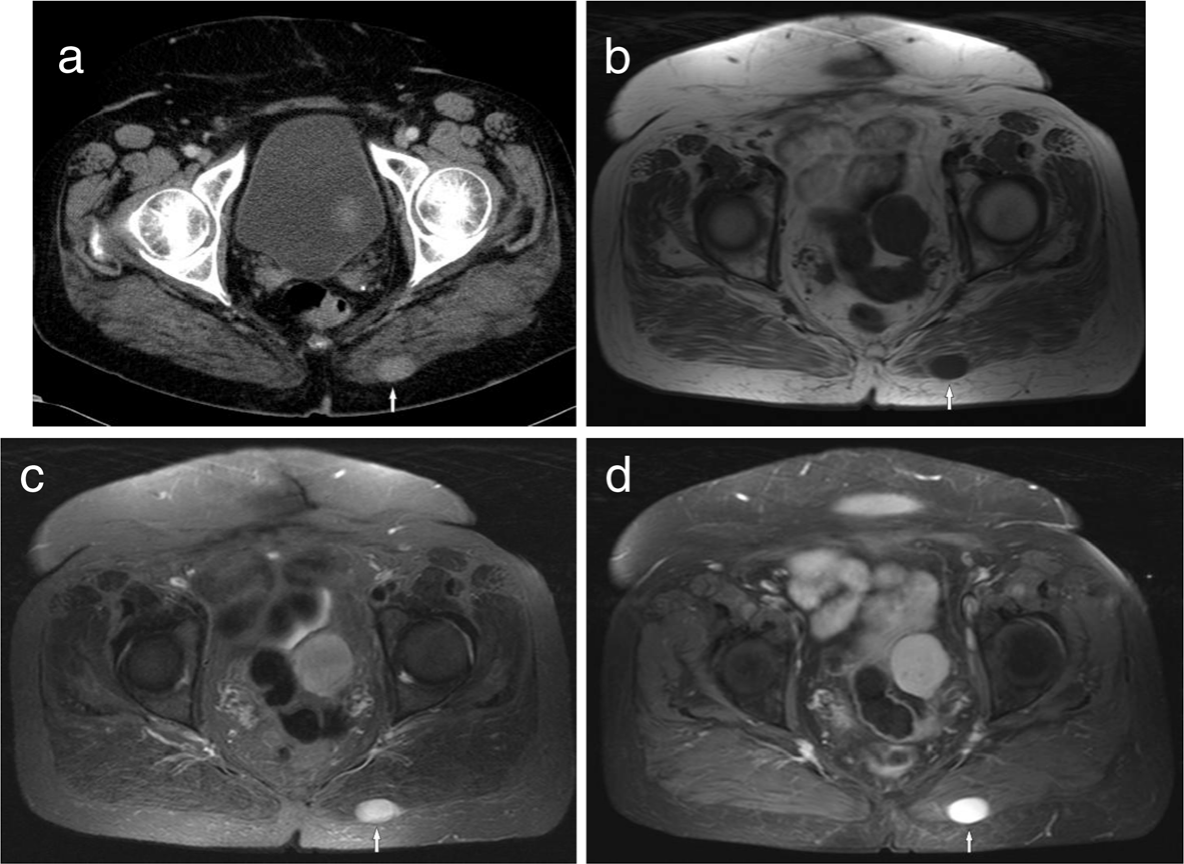
**Imaging findings in a 50 year old woman with known history of melanoma. (a)** CT image showing an oval mass with homogeneous contrast enhancement (type I) in the left gluteal musculature. **(b)** The lesion is hypointense on T1w image (spin echo pulse sequence, TR/TE: 569/11 ms). **(c)** The lesion is hyperintense on T2w image (short tau inversion recovery (STIR), TR/TE: 5210/80 ms). **(d)** After intravenous administration of contrast medium the lesion shows marked homogeneous enhancement (T1w spin echo with fat saturation, TR/TE: 610/12 ms).

**Figure 2:**
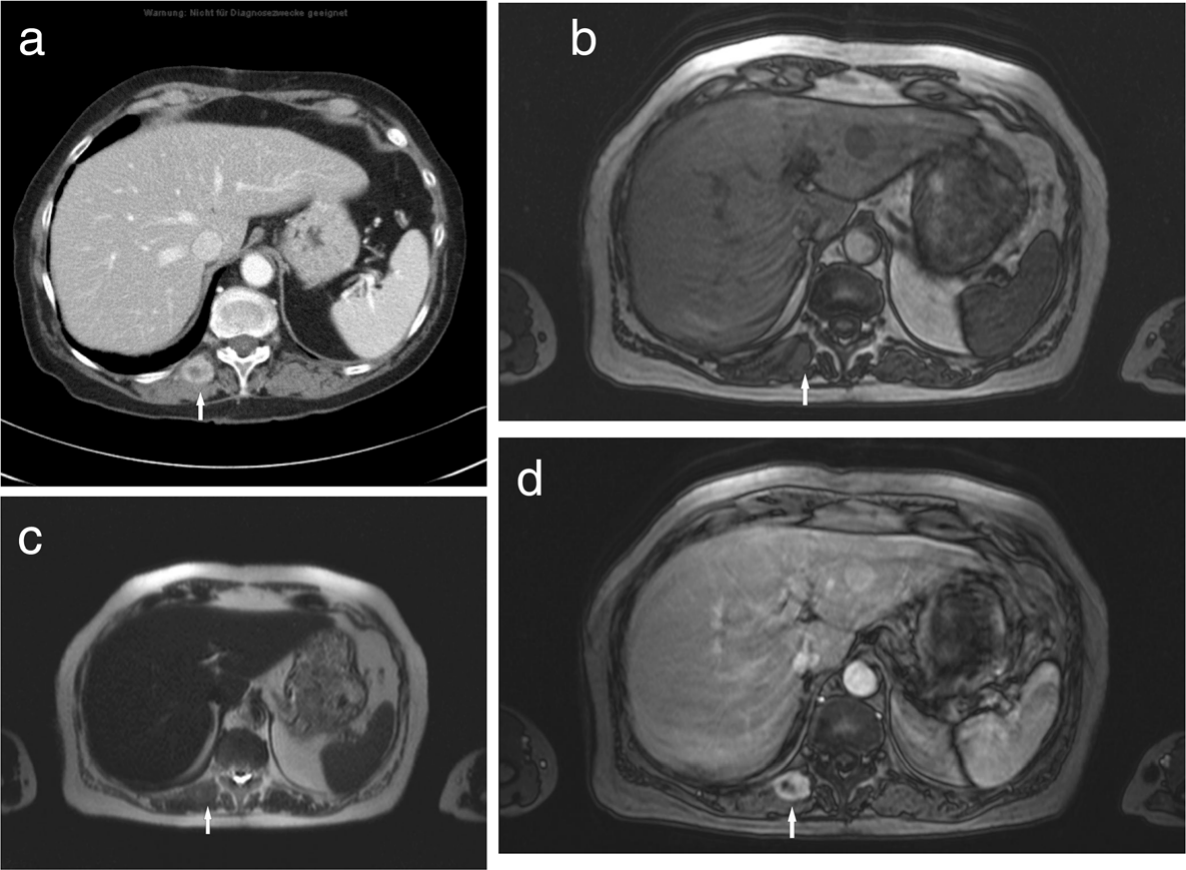
**Type II metastasis in the right paravertebral musculature in a patient with lung carcinoma. (a)** On CT, the lesion (arrow) shows central low attenuation and rim enhancement. **(b)** On the T1w image (T1w flash 2D, TR/TE: 142/2.2 ms) the lesion (arrow) is isointense in comparison to the unaffected musculature. **(c)** On T2w image (half-Fourier acquisition turbo spin echo pulse sequence, HASTE, TR/TE: 800/120 ms) the lesion is isointense (arrow). **(d)** On MRI after administration of contrast medium (T1w flash 2D image with fat saturation, TR/TE: 209/2.3 ms) the lesion shows central low attenuation and rim enhancement (arrow).

**Figure 3:**
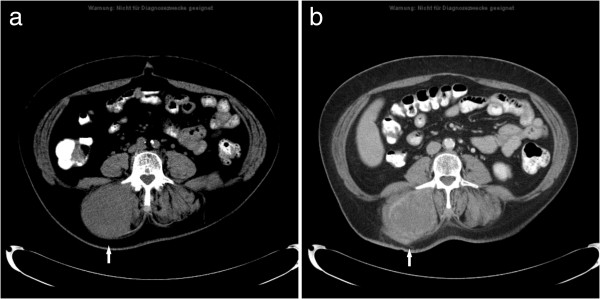
**MM type III in a patient with known metastatic soft tissue sarcoma.** CT images before **(a)** and after administration of contrast medium **(b)** documenting a massive hypodense enlargement of the right paravertebral musculature with inhomogeneous enhancement (arrow).

**Figure 4:**
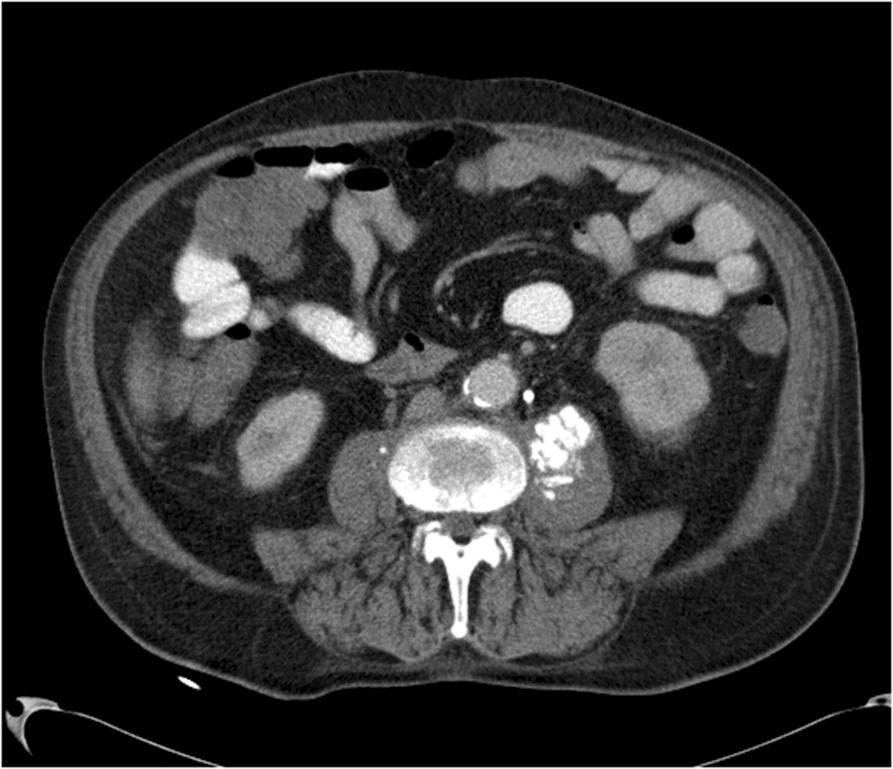
**MM type IV in a patient with urinary bladder carcinoma.** CT image demonstrates multiple calcifications in the left iliopsoas muscle (arrow).

**Figure 5:**
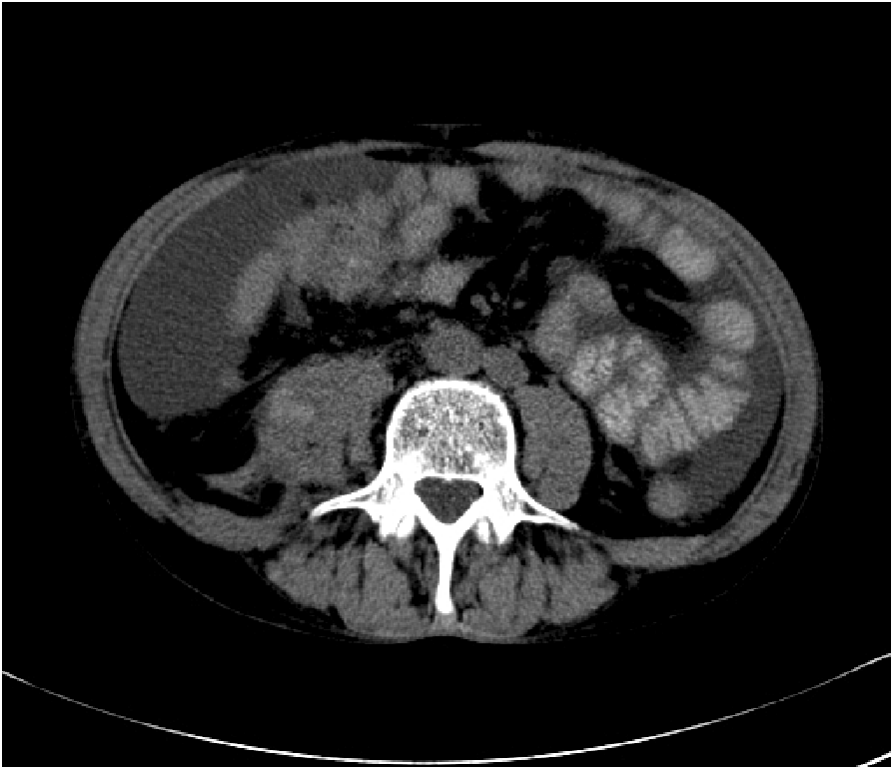
**MM type V in a patient with known history of esophageal cancer.** CT without intravenous administration of contrast medium shows hyperdense areas (arrow) in the right iliopsoas muscle.

CT images were available for 260 metastases. On these images, five different patterns of MM were found. 46.5% presented as round or oval masses with homogeneous contrast enhancement (type I, Figure 1). In 27.7% of MM type II was diagnosed. These metastases manifested as abscess-like intramuscular lesions with central low attenuation and rim enhancement (Figure 2). Type III of MM presented as diffuse infiltration with muscle swelling and inhomogeneous enhancement (Figure 3) and was seen in 18.1%. Type IV of MM showing multiple intramuscular calcifications (Figure 4) occurred in 6.5%. 1.2% of MM presented as intramuscular bleeding (type V, Figure 5).

CT features of MM arising from frequent primary tumours are listed in Table 4. Type II lesions occurred significantly more often in lung cancer than in stomach cancer (p = 0.042), breast carcinoma (p = 0.021), and renal cell carcinoma (p = 0.02). MM from renal cell carcinoma presented more often as type I lesions in comparison with urothel carcinoma (p = 0.042). Type IV MM were seen more often in stomach cancer than in lung cancer (p = 0.042).

**Table 4:** Comparison of CT features of MM in frequent primary tumours (more than 15 lesions)

**Primary tumours**	**CT-Type**				
	**Type I**	**Type II**	**Type III**	**Type IV**	**Type V**
	**n (%)**	**n (%)**	**n (%)**	**n (%)**	**n (%)**
**Lung cancer, LC (n = 45)**	13 (28.9)	23 (51.1) p = 0.042 vs SC	8 (17.8)	1 (2.2) p = 0.042 vs SC	
**Colonic cancer, CC (n = 27)**	9 (33.3)	9 (33.3)	5 (18.5)	3 (11.1)	1 (3.7)
**Stomach cancer, SC (n = 17)**	7 (41.2)	1 (5.9)	5 (29.4)	4 (23.5)	
**Breast carcinoma, BC (n = 26)**	16 (61.5)	1 (3.8) p = 0.021 vs LC	9 (34.6)		
**Malinant melanoma, MMe (n = 16)**	9 (56.3)	5 (31.3)	2 (12.5)		
**Renal cell carcinoma, RCC (n = 22)**	19 (86.4) p = 0.042 vs UC	2 (9.1) p = 0.02 vs LC	1 (4.6)		
**Urothel carcinoma, UC (n = 16)**	4 (25.0)	6 (37.5)	4 (25.0)	2 (12.5)	

The median size as determined by measuring the maximum diameter of the MM that presented as masses was 44.9 ± 35.9 mm. There were no significant differences between the sizes of MM depending on the primary tumour.

### MRI features of MM

MRI findings were available for 98 MM. On T2W images 81.6% of the metastases were hyperintense in comparison to unaffected musculature, 9.2% were mixed iso- to hyperintense, 6.1% isointense, and 3.1% metastases were hypointense. On T1W images 48.3% of the MM were homogeneously isointense compared with unaffected muscle tissue, 31.9% were hyperintense, and 19.8% were hypointense.

**Figure 6:**
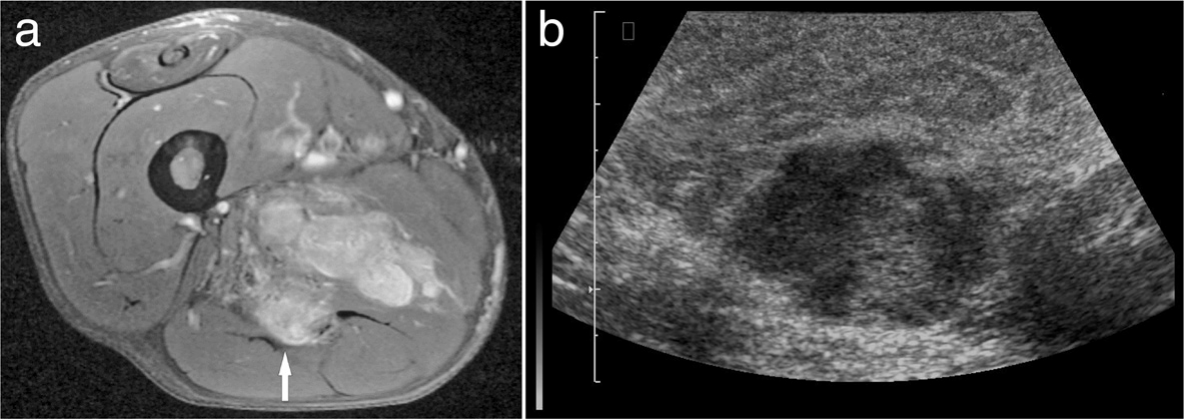
**MM in the thigh musculature in a patient with known history of metastatic renal cell carcinoma. (a)** T1w image (turbo spin echo sequence with fat saturation, TR/TE: 615/11 ms) after contrast administration shows a lobulated mass (arrow) with inhomogeneous enhancement (arrow). On ultrasound, the mass was mixed hypo- to hyperechoic in comparison to the unaffected muscle **(b)**.

After intravenous administration of contrast medium most lesions (87.2%) showed a heterogeneous enhancement. Homogeneous enhancement was seen in 12.8% of the cases (Figures 1d, 6a). There were no differences in MRI features of MM in different primary tumours.

### Ultrasound findings of MM

US features of 40 lesions were available. 39 MM (97.5%) were hypoechoic and one metastasis (2.5%) was hyperechoic (Figure 6b). Because of the small number of MM investigated by US no further statistical analysis was performed.

### PET images of MM

**Figure 7:**
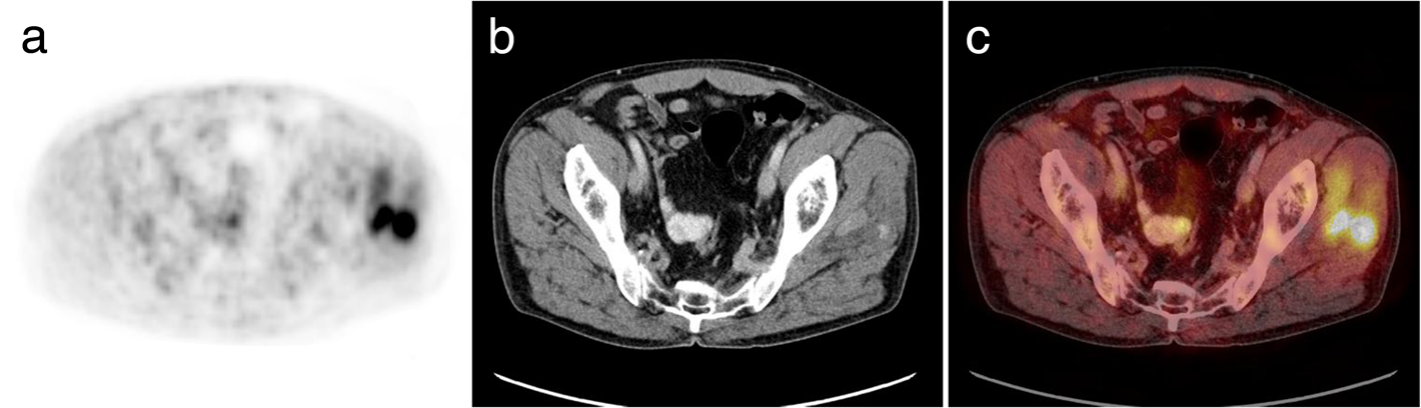
**Imaging findings in a patient with lung cancer.** PET **(a)** showing focal uptake in the left gluteal musculature (arrow). CT scan **(b)** detecting intramuscular masses in this area (arrow). Fusion image **(c)**.

PET features of 28 patients (40 lesions) were available. There were 22 patients with lung cancer, 9 patients with esophageal cancer, 2 patients with gall bladder carcinoma, 2 patients with renal cell carcinoma, 1 patient with sarcoma, 1 patient with carcinoma of cervix uteri and 1 patient with endometrial cancer. All identified MM presented as focally abnormal intramuscular uptake (Figure 7) with SUV ranging from 2.4 to 20.3, median SUV 6.7 (mean SUV = 8.0 ± 4.4). Because of the small number of MM in different primary tumours investigated by PET or PET/CT no further statistical analysis was performed.

## Discussion

Previously, some meta-analyses regarding MM were reported [18-20]. In these publications primary tumours, prevalence of MM and their localisations were described. The number of reported lesions was up to 254 [18-20]. Our report with 461 patients/682 lesions is the largest to date. Furthermore, this is the first analysis of radiological patterns of MM in dependency on primary tumours.

According to Haygood et al., most common primary malignancies were lung cancer, sarcomas, melanoma, renal cell carcinoma and breast cancer in decreasing order of frequency [18]. In a previously reported mono-center study, MM from urogenital tumours occurred most commonly, followed by gastrointestinal tumours and malignant melanoma [6]. In the present analysis, lung cancer, gastrointestinal tumours, urogenital tumours, and breast cancer were the most frequent primary malignant diseases.

In previous reports, most MM were localised in the trunk musculature, lower extremities and in the gluteal muscles [18]. Our results showed that MM were localised most frequently in the thigh muscles, extraocular musculature, gluteal and paravertebral muscles. Furthermore, we found that several primary malignancies showed different MM localisations. For example, lung cancer tends to metastasise to the extremities, whereas most MM from breast cancer were located in the extraocular musculature. Urothel carcinomas metastasise significantly more often into the iliopsoas musculature. This finding may be related to the fact that the primary tumours have different metastatic routes. Furthermore, it must be presumed that they have different pathophysiological mechanisms of intramuscular metastatic spread.

According to the literature, there are three important pathophysiological mechanisms. Firstly, MM can develop via the arterial route [4,6]. Secondly, malignant tumours can metastasise into the musculature via venous vessels, especially through the paravertebral venous plexus [21]. Paravertebral veins have multiple connections to the inferior vena cava and the mesenterial venous system. As reported previously, pelvic and abdominal malignancies metastasise often via the paravertebral veins [21]. Thirdly, MM can originate in intramuscular aberrant lymph nodes, especially MM in the psoas muscle [22].

On CT, the most frequent findings were intramuscular lesions with homogeneous enhancement (type I). As reported previously, these metastatic lesions should be differentiated from several benign diseases, such as muscle hemangioma, intramuscular ganglion, and myxoma [23,24].

Lesions with central low attenuation and rim enhancement were seen in 27.7% of MM. These lesions can be mistaken for intramuscular abscesses [6]. However,secondary abscess formation in intramuscular metastases has also been described [6].

Diffuse metastatic muscle infiltration was seen in 18.1% of MM. This pattern can be misdiagnosed as muscle sarcoma or primary/secondary muscle lymphoma [25,26].

6.6% of MM manifested as intramuscular calcifications. The cause of this neoplasm-induced intramuscular ossification is unknown. These MM can mimic benign muscle calcifications, which occurs in myositis ossificans, intramuscular angiomatosis, systemic sclerosis, and calcific myonecrosis [27,28].

1.2% of MM presented with intramuscular bleeding. This pattern of MM has been described only sporadically by now [6,29].

As seen, MM can manifest with a broad spectrum of radiological features. Furthermore, we hypothesize that several malignancies might produce different metastatic patterns in the musculature. In fact, type II lesions occurred significantly more often in lung cancer than in stomach cancer, breast carcinoma, or renal cell carcinoma. In contrast to other tumours, MM from stomach cancer tend to manifest as diffuse muscle infiltration.

On MRI, most lesions were hyperintense on T2w and hypointense on T1w in comparison to unaffected musculature, with heterogeneous contrast enhancement. This finding is in agreement with previous reports [5,12,15,17]. We found no differences in MRI features of MM between different primary tumours.

Previously, US findings of MM have been reported only in isolated case reports. Our analysis shows that on US most MM were hypoechoic.

On PET/CT, MM manifested as focal hypermetabolic lesions. The finding corresponds well with those of other authors [30-32]. Again, there were no differences of PET/CT features of MM with differentprimary malignancies.

Our study has several limitations. It is retrospective, and most MM were acquired from the literature. Some primary tumours/MM could not be included into the statistical analysis because of the small number of patients/lesions. Furthermore, not every patient/lesion was investigated by all radiological methods i.e. CT, MRI, US, and PET/CT. These limitations can explain that only for CT specific radiological features could be associated with different primary tumours.

## Conclusion

Our study shows that MM present with a broad spectrum of radiological features.

CT findings of MM show differences between different primary tumours. The localisation of MM also varies with different primary malignancies.

## Competing interests

The authors declare that they have no competing interests.

## Authors’ contributions

Study concepts: AS, RPS. Study design: AS. Data acquisition: JK, AS, AGB, DS. Quality control of data and algorithms: AS, AGB, DS, CB. Data analysis and interpretation: AS, AGB, CB. Statistical analysis: AS, JK, AW. Manuscript preparation: AS, JK, AGB, DS, CB. Manuscript editing: RPS. Manuscript review: AS, RPS. All authors read and approved the final manuscript.

## References

[B1] Acinas Garcia O Fernandez FA Satue EG Buelta L Val-Bernal JF Metastasis of malignant neoplasms to skeletal muscle Rev Esp Oncol 1984 31 57 67 6545428

[B2] Hasegawa S Sakurai Y Imazu H Matsubara T Ochiai M Funabiki T Suzuki K Mizoguchi Y Kuroda M Kasahara M Metastasis to the forearm skeletal muscle from an adenocarcinoma of the colon: report of a case Surg Today 2000 30 1118 1123 10.1007/s005950070013 11193747

[B3] Nabeyama R Tanaka K Matsuda S Iwamoto Y Multiple intramuscular metastases 15 years after radical nephrectomy in a patient with stage IV renal cell carcinoma J Orthop Sci 2001 6 189 192 10.1007/s007760100070 11484108

[B4] Willis RA The Spread of Tumours in the Human Body 1952 London: Butterworth

[B5] Glockner DM White LM Sundaram M McDonald DJ Unsuspected metastases presenting as solitary soft tissue lesions: a fourteen-year review Skeletal Radiol 2000 29 270 274 10.1007/s002560050606 10883446

[B6] Surov A Hainz M Holzhausen HJ Arnold D Katzer M Schmidt J Spielmann RP Behrmann C Skeletal muscle metastases: primary tumours, prevalence, and radiological features Eur Radiol 2010 20 649 658 10.1007/s00330-009-1577-1 19707767

[B7] Djaldetti M Sredni B Zigelman R Verber M Fishman P Muscle cells produce a low molecular weight factor with anti-cancer activity Clin Exp Metastasis 1996 14 189 196 8674272 10.1007/BF00053891

[B8] Bar-Yehuda S Barer F Volfsson L Fishman P Resistance of muscle to tumor metastases: a role for A3 adenosine receptor agonists Neoplasia 2001 3 125 131 10.1038/sj.neo.7900138 11420748 PMC1505413

[B9] Weiss L Biomechanical destruction of cancer cells in skeletal muscle: a rate-regulator for hematogenous metastasis Clin Exp Metastasis 1989 5 483 491 2752602 10.1007/BF01753809

[B10] Seely S Possible reasons for high resistance of muscle to cancer Med Hypotheses 1980 6 133 137 10.1016/0306-9877(80)90079-1 7393016

[B11] Magee T Rosenthal H Skeletal muscle metastases at sites of documented trauma AJR Am J Roentgenol 2002 178 985 988 10.2214/ajr.178.4.1780985 11906887

[B12] Tuoheti Y Okada K Osanai T Tuoheti Y Okada K Osanai T Nishida J Ehara S Hashimoto M Itoi E Skeletal muscle metastases of carcinomas: a clinicopathological study of 12 cases Jpn J Clin Oncol 2004 34 210 214 10.1093/jjco/hyh036 15121758

[B13] O’Brien JM Brennan DD Taylor DH Holloway DP Hurson B O'Keane JC Eustace SJ Skeletal muscle metastasis from uterine leiomyosarcoma Skeletal Radiol 2004 33 655 659 10.1007/s00256-004-0787-5 15127247

[B14] Pretorius ES Fishman EK Helical CT of skeletal muscle metastases from primary carcinomas AJR Am J Roentgenol 1999 174 401 404 10658714 10.2214/ajr.174.2.1740401

[B15] Lee BY Choi JE Park JM Jee WH Kim JY Lee KH Kim HS Song KS Various image findings of skeletal muscle metastases with clinical correlation Skeletal Radiol 2008 37 923 928 10.1007/s00256-008-0510-z 18594814

[B16] Williams JB Youngberg RA Bui-Mansfield LT Pitcher JD MR imaging of skeletal muscle metastases AJR Am J Roentgenol 1997 168 555 557 10.2214/ajr.168.2.9016246 9016246

[B17] Surov A Fiedler E Voigt W Wienke A Holzhausen HJ Spielmann RP Behrmann C Magnetic resonance imaging of intramuscular metastases Skeletal Radiol 2011 40 439 446 10.1007/s00256-010-1018-x 20697708

[B18] Haygood TM Wong J Lin JC Li S Matamoros A Costelloe CM Yeung H Sandler CM Nunez RF Kumar R Madewell JE Skeletal muscle metastases: a three-part study of a not-so-rare entity Skeletal Radiol 2011 41 899 909 22101865 10.1007/s00256-011-1319-8

[B19] Damron T Heiner J Distant soft tissue metastases: a series of 30 new patients and 91 cases from the literature Ann Surg Oncol 2000 7 526 534 10.1007/s10434-000-0526-7 10947022

[B20] Herring CL Jr Harrelson JM Scully SP Metastatic carcinoma to skeletal muscle. A report of 15 patients Clin Orthop Relat Res 1998 355 272 281 9917613 10.1097/00003086-199810000-00029

[B21] Vider M Maruyama Y Narvaez R Significance of the vertebral venous (Batson’s) plexus in metastatic spread in colorectal carcinoma Cancer 1977 40 1 67 71 10.1002/1097-0142(197707)40:1<67::AID-CNCR2820400113>3.0.CO;2-F 880573

[B22] Lee JK Glazer HS Psoas muscle disorders: MR imaging Radiology 1986 160 3 683 687 3737906 10.1148/radiology.160.3.3737906

[B23] Prado MA Miró RL Leal IM Vargas J Dorrego EJ Intramuscular myxoma: differential diagnosis and review of the literature Orthopedics 2002 25 11 1297 1299 12452352 10.3928/0147-7447-20021101-26

[B24] Martin S Rapariz JM Osés MJ Martinez C A possible cause of multiple intramuscular masses: Mazabraud’s syndrome Eur Radiol 2007 18 2 417 421 18246358 10.1007/s00330-007-0653-7

[B25] Eustace S Winalski CS McGowen A Lan H Dorfman D Skeletal muscle lymphoma: observation at MR imaging Skeletal Radiol 1996 25 425 430 10.1007/s002560050110 8837273

[B26] Suresh S Saifuddin A O’Donnell P Lymphoma presenting as a musculoskeletal soft tissue mass: MRI findings in 24 cases Eur Radiol 2008 18 1628 1634 10.1007/s00330-008-1014-x 18493781

[B27] Geukens DM Vande Berg BC Malghem J De Nayer P Galant C Lecouvet FE Ossifying muscle metastases from an esophageal adenocarcinoma mimicking myositis ossificans AJR Am J Roentgenol 2001 176 1165 1166 10.2214/ajr.176.5.1761165 11312174

[B28] McCarthy EF Sundaram M Heterotopic ossification: a review Skeletal Radiol 2005 34 609 619 10.1007/s00256-005-0958-z 16132978

[B29] Baud O Dubost JJ Soubrier M Boisgard S Verelle P Sauvezie B Recurrent posttraumatic hematoma leading to discovery of a muscle metastasis Rev Rhum Engl Ed 1997 64 6 433 9513621

[B30] Khandelwal AR Takalkar AM Lilien DL Ravi A Skeletal muscle metastases on FDG PET/CT imaging Clin Nucl Med 2012 37 575 579 10.1097/RLU.0b013e3182443e32 22614189

[B31] Surov A Pawelka MK Wienke A Schramm D PET/CT imaging of skeletal muscle metastases Acta Radiol 2014 55 1 101 106 10.1177/0284185113493086 23884841

[B32] Purandare NC Rangarajan V Pramesh CS Rajnish A Shah S Dua SG Isolated asymptomatic skeletal muscle metastasis in a potentially resectable non-small cell lung cancer: detection with FDG PET-CT scanning Cancer Imaging 2008 8 216 219 10.1102/1470-7330.2008.0033 19042177 PMC2590881

